# Crosstalk of NPY and TGFβ orchestrates the signaling to facilitate perineural invasion of oral squamous cell carcinoma

**DOI:** 10.1038/s41416-025-03261-5

**Published:** 2025-11-26

**Authors:** Jing Bi, Ketong Liu, Yiru Luo, Yueqi Zhou, Zhengyan Liu, Junting Tao, Jinhui Wei, Marene Landstrom, Yabing Mu, Guangxiang Zang

**Affiliations:** 1 Liaoning Provincial Key Laboratory of Oral Disease, School and Hospital of Stomatology, China Medical University, Shenyang City, P.R. China; 2 Department of Medical Bioscience, Building 6M, Umeå University, Umeå, Sweden

**Keywords:** Oral cancer detection, Cell migration, Tumour biomarkers

## Abstract

**Background:**

Perineural invasion (PNI) frequently occurs in oral squamous cell carcinoma (OSCC) and predicts poor prognosis. Although PNI is increasingly recognised as a process driven by tumour-nerve crosstalk, the underlying molecular mechanisms remain unclear. We investigated the role of sympathetic nerve–derived neuropeptide Y (NPY) and its receptor NPY1R in OSCC PNI.

**Methods:**

NPY/NPY1R expression was assessed in human OSCC tissues by immunostaining, qPCR, and TCGA data analysis. Functional studies using Cal27 and SCC9 cells included migration, invasion, and sphere assays. The causal role of NPY1R was tested by lentiviral knockdown/overexpression, validated in tongue orthotopic xenografts, and further examined by NPY1R pharmacological inhibition in vivo.

**Results:**

NPY was enriched in the PNI microenvironment, and malignant OSCC expressed high NPY1R, particularly at invasive fronts. Mechanistically, NPY activated ERK and Smad2 via NPY1R, synergising with TGFβ signalling in tumour cells expressing TβRI. This crosstalk enhanced proliferation, invasion, and PNI in vivo. Importantly, NPY1R inhibition markedly reduced tumour growth, metastasis, and PNI.

**Conclusions:**

We identify NPY-NPY1R-TGFβ crosstalk as a novel mechanism enabling OSCC to exploit neural signals for PNI, highlighting a promising therapeutic target to block neural invasion and improve patient outcomes.

## Introduction

Oral squamous cell carcinoma (OSCC), the most prevalent malignancy in the head and neck region, frequently exhibits perineural invasion (PNI), a pathological process wherein tumour cells infiltrate along peripheral nerves. Due to the dense innervation of the oral cavity, PNI is observed over 80% of OSCC cases, which is strongly correlated with increased recurrence, metastasis, and poor patient survival, often necessitating aggressive adjuvant therapies after surgery [1–3].

PNI represents a unique tumour-nerve interactive microenvironment, where reciprocal communication occurs between neoplastic and neural components. Peripheral nerves actively secrete various signalling molecules including neuropeptides, neurotransmitters, and nerve growth factors, to modulate tumour progression. In turn, tumour cells release mediators that influence axonogenesis, neurogenesis, and neurotropism, facilitating neural remodelling and invasion [4–6]. Although recent studies have begun to explore the bidirectional nature of this interaction, the dynamic molecular crosstalk that governs PNI remains largely uncharacterised.

Neuropeptides, a structurally diverse group of signalling molecules, play pivotal roles in numerous physiological processes. Unlike classical neurotransmitters, neuropeptides act over longer distances and durations due to their slower degradation and absence of rapid reuptake mechanisms. Their receptors, typically G protein-coupled receptors (GPCRs), exhibit high affinity and sensitivity, allowing neuropeptides exert potent biological effects even at low concentrations over relatively longer distances [7, 8]. Among neuropeptides, neuropeptide Y (NPY) is well-characterised in the central and peripheral nervous system, where it regulates appetite, metabolism, stress response, and nociception [9–11]. NPY receptors are even more widely distributed, and emerging evidence implicates NPY and its receptors in cancer progression. NPY can activate ERK1/2 MAPK pathway via Y1R, Y2R, or Y5R receptors to promote tumour cell proliferation, and enhances the motility of various cancer cells. In addition, NPY plays a key role in modulating the tumour microenvironment and altering cellular energetics [12, 13]. Despite these insights, the role of NPY signalling in perineural invasion, particularly in OSCC remains largely unexplored.

Herein, we aim to elucidate the role of NPY/NPY1R signalling within the PNI microenvironment of OSCC. We found that NPY not only activates ERK signalling, but also enhances TGFβ canonical Smad2 signalling, thereby promoting PNI. Our findings uncover a previously unrecognised neural-tumour axis mediated by NPY that facilitates neural invasion in OSCC. This study advances our understanding of the molecular mechanisms underlying PNI and highlights the NPY/NPY1R-TGFβ signalling pathway as a promising therapeutic target for aggressive OSCC.

## Materials and methods

### Collection of OSCC tissue

Human OSCC tumour tissues and the adjacent normal epithelial tissues were collected from surgical specimens in the Stomatology Hospital, China Medical University (Shenyang, China). The human specimens involved in our study were reviewed and approved by the Ethics Committee of Stomatology Hospital, China Medical University. All the experiments were undertaken following the ethics permission (G2020004), following the Declaration of Helsinki. Informed consent was obtained from all patients. Two pathologists in Stomatology Hospital, China Medical University confirmed the diagnosis of OSCC.

### Analysis of PNI

The nerve identification and analysis in OSCC in a cohort comprising 103 patients, followed the methods described by Schmitd.L. et al. previously [14]. The H&E slices were scanned with the Motic DSAssistant (4 K), and the slices were fully measured with a repeat measurement. PNI-positive cases were identified as the tumours close to the nerve and involved at least 33% of the nerve circumference or the tumour cells located in any of the three layers of the nerve sheath; otherwise, PNI-negative cases were identified. Using the scanned image, nerve-tumour distance and the diameter of each nerve were measured. Kaplan–Meier methods were used to estimate disease-specific survival (DSS) probabilities within groups, using the time of diagnosis as the baseline when defining DSS.

### Antibodies and reagents

The primary antibodies: NPY(ab221145, Abcam), NPY1R (ab91262, Abcam), βIII tubulin (ab18207, Abcam), S100 (IR50461, Dako), TβRI (ab235578, Abcam), β-actin (ab8227, Abcam), TGFβ1 (ab215715, Abcam), pSmad2 (3108 L, Cell Signalling), Smad2 (ab71109, Abcam), pERK (4370S, Cell Signalling), ERK (4696S, Cell Signalling), E-Cadherin (610182, BD bioscience), PGP9.5 (ab8189, Abcam), Pan cytokeratins (IHCR2025-6, Sigma), Ki67 (ab15580, Abcam).The secondary antibodies of Alexa Fluor 488 (A-21206, Invitrogen), Alexa Fluor 555 (A-31570, Invitrogen), and Hoechst (H1399, Invitrogen) were used for immunofluorescence staining. The HRP-conjugated Goat Anti Rabbit IgG (BL003A, Biosharp), and Goat Anti Mouse IgG (BL001A, Biosharp) were used for Western blotting.

### Immunohistochemistry (IHC) and immunofluorescence (IF) staining

As reported previously [15, 16], human tissue and OSCC sphere cultured in the Matrigel were fixed in 4% paraformaldehyde, embedded in paraffin, and sectioned for H&E, IHC and IF staining. Antigen retrieval and IHC staining followed the staining kit protocol (GK600705, GeneTech). Images were taken with Olympus BX53 microscope and analyzed by Image J software. The data was assessed by other researchers.

IHC staining was scored by evaluating both the percentage of positive cells and staining intensity [17]. The percentage of positive cells was graded at four levels: 0 (0–4%), 1 (5–25%), 2 (26–50%), and 3 (51–100%). The staining intensity was graded as 1 (weak, light brown), 2 (moderate, brown), or 3 (strong, dark brown). The final immunoreactivity score was calculated as the sum of the percentage score and the intensity score. Based on this scoring system, cases with a total score <4 were defined as having low expression, whereas cases with a score ≥4 were defined as having high expression.

For IF staining, the sections were incubated with primary antibodies overnight at 4 °C, second antibodies for 45 min at room temperature, co-stained with Hoechst (H1399, Invitrogen), and mounted with Flourmountain-G (0100-01, Southern Biotech). Images were taken with a confocal microscope (Olympus, FV3000).

### RNA isolation and qRT-PCR analysis

The total RNA of the tissues or cells was extracted using TRIZOL (Invitrogen) according to the manufacturer’s protocol. 1 µg RNA was used for cDNA synthesis using Hifair III Strand cDNA Synthesis SuperMix (11141ES60, YEASEN). qRT-PCR detection was performed using Hieff qPCR SYBR Green Master (Low ROX, 11202ES08, YEASEN). The results were represented as fold change using ΔΔCt method, and each experiment was repeated at least three times. The primers were as follows:

NPY1R F:GAGGCGATGTGTAAGTTGAATC; R:ACCCAAATCACAGCAATACCTA.

GAPDH F:GACAGTCAGCCGCATCTTCT; R:TTAAAAGCAGCCCTGGTGAC.

### Public TCGA data and bioinformatic analysis

HNSCC’s transcriptomic data were obtained from the Cancer Genome Atlas (TCGA) [18]. R package “ggplot2” was used to display an overview of the expression of NPY, NPY1R, and NPY5R. Statistical analysis and visualisation were carried out in R 3.6.3. Kruskal-Wallis Test and Dunn’s Test were used for statistics.

The differentially expressed genes (DEGs) between the PNI-positive (n = 182) and PNI-negative (n = 202) samples in TCGA-HNSCC were identified by R package limma via generalised linear models. The genes (n = 326) with a false-discovery rate (FDR) ≤ 0.05 were considered. These 326 DEGs were performed enrichment analysis in KEGG pathways by Enrichr (https://maayanlab.cloud/Enrichr/). The top 10 pathways with the lowest FDR were selected as the most significant pathways.

### Cell culture

OSCC cell lines Cal27 and SCC9 were used in this study and authenticated using STR profiling. HDMEM media is for Cal27, and RPMI1640 is for SCC9, supplied with 10% FBS and 1% penicillin/streptomycin (Gibco). The cells were cultured in the humidity incubator at 37 °C, 5% CO_2_, and maintained in mycoplasma-free condition. The cells were starved for 12–18 h in a medium supplemented with 0.1% FBS before NPY stimulation, and in a medium supplemented with 1% FBS before TGFβ1 stimulation respectively. Inhibitors were used one hour before stimulation. NPY (90880-35-6, Bio-techne) at 1 ng/ml, NPY1R antagonist BIBO3304 (2412, TOCRIS) at 0.2 µM. TGFβ1 (885-GS, R&D Systems) at 5 ng/ml, TβRI kinase inhibitor LY2157299 (HY-15150, MedChem Express) at 1 µM.

### Western blotting detection

Sample lysates were prepared using RIPA lysis buffer (P0013B, Beyotime), and the protein concentration was measured by BCA protein assay (P0010, Beyotime). Equal amounts of samples were loaded into the Tris-glycine gel (P0012AC, Beyotime), and transferred to polyvinylidene fluoride membrane (Merck KGaA, Darmstadt). The membrane was blocked with 5% milk in TBST, incubated with the primary antibodies overnight at 4 °C and the second antibodies for 1 h, and detected using the Enhanced ECL kit (36222ES60, YEASEN). Densitometry analysis was performed with Tanon 5200.

### Cell Counting Kit-8 (CCK-8) assay

Cell viability was assessed by Cell Counting Kit-8 (CCK-8) assay according to the manufacturer’s guidance (Beyotime, China). 2000 tumour cells were seeded in 96-well plates, cultured overnight, and then treated with NPY for the indicated time. Finally, the cells were incubated with CCK-8 staining buffer for one h at 37 °C, and the optical density (O.D.) at 450 nm was measured by Multiscan FC Microplate Photometer (Thermo Scientific).

### 3D sphere culture

As reported previously [15, 16], 2000 tumour cells mixed with 20 µL Matrigel (356230, Corning) were seeded on the coverslip, incubated at 37 °C for 30 min to solidify the Matrigel, and then cultured in the medium at 37 °C, 5% CO_2_. Morphological features of the spheres were observed with an inverted microscope (Leica Microsystems DFC550).

### Colony assay

500 tumour cells were seeded in 6-well plates and cultured in 10% FBS medium for 2 weeks to grow into colonies. The colonies were fixed using 4% PFA and then stained with crystal violet for 10 min. After washing, the colonies were scanned (V850 pro, Epson).

### Scratching assay

Tumour cells were seeded in 6-well plates and cultured until confluency, and the scratching was made with 200 µL pipette tips. These cells were stimulated with or without NPY, TGFβ, in a condition with or without NPY1R inhibitor, or TβRI inhibitor for the indicated time. Morphological features were observed under the inverted microscope.

### Transwell invasion assay

1 × 10^5^ tumour cells were seeded in the upside chamber of transwell (Corning, USA) with 1% FBS medium, and the lower chamber had 5% FBS medium. The tumour cells were stimulated with or without NPY, TGFβ, in a condition with or without NPY1R inhibitor, or TβRI inhibitor for 36 h. Non-invasive cells were removed from the upside of the chamber, and the invasive cells were stained with 10% crystal violet and photographed with Olympus microscope (BX53, Olympus). Colorimetric quantification was performed after the membrane was inserted into the extraction solution for 10 min. Optical Density (O.D.) at 560 nm was measured by Multiscan FC Microplate Photometer (Thermo Scientific).

### Lentivirus-based knockdown or overexpression

Purified lentiviral particles of pLV-NPY1R-EGFP, NPY1R shRNA (pLV-shRNA-EGFP), and negative control lentiviral particles (pLV-NC-EGFP) were purchased from Shanghai Genepharma Biotechnology. Following the protocol described before for the lentivirus-based gene delivery system [19], 50–60% confluence cells were transfected with viral particles for 48 h. Then, puromycin (4 μg/mL) was used to select the stably transfected cells for further experiment.

### Orthotopic tongue xenograft model

All animal experiments were conducted in accordance with protocols approved by the Animal Care Committee of China Medical University (approval number: CMU2023684). Six-week-old male NTG mice (SPF Biotechnology, China) were randomly assigned into experimental groups and anaesthetised with 2% isoflurane. A total of 1 × 10^5^ tumour cells were injected beneath the lingual mucosa to establish orthotopic tongue xenografts. Body weight and tumour size were monitored every two days. Animals were euthanized after four weeks of treatment or earlier if body weight loss exceeded 20–25% of baseline. For the inhibitor treatment, mice received intraperitoneal injections of 200 µL of the NPY1R inhibitor BIBO3304 (100 µg/kg), or 200 µL PBS in the control group on alternate days on the same schedule. Tumours and sentinel lymph nodes were harvested, sectioned, and processed for protein extraction, RNA isolation, and histological analysis following standard protocols, performed by other researchers.

### Statistical analysis

Data are presented as mean ± standard deviation (SD), and each experiment was repeated at least thrice. GraphPad Prism version 8 (GraphPad Software, San Diego, CA, USA) was used to analyze raw data and plot results. The normality of all data was assessed and Data were analyzed by two-tailed unpaired Student’s *t* test (two groups) or one-way ANOVA (multiple comparisons), unless otherwise noted. Significance was denoted as follows: * *P* < 0.05, ** *P* < 0.01, *** *P* < 0.001; not significant (n.s.).

## Results

### NPY1R is expressed in PNI and correlated with malignancy of OSCC

**Fig. 1:**
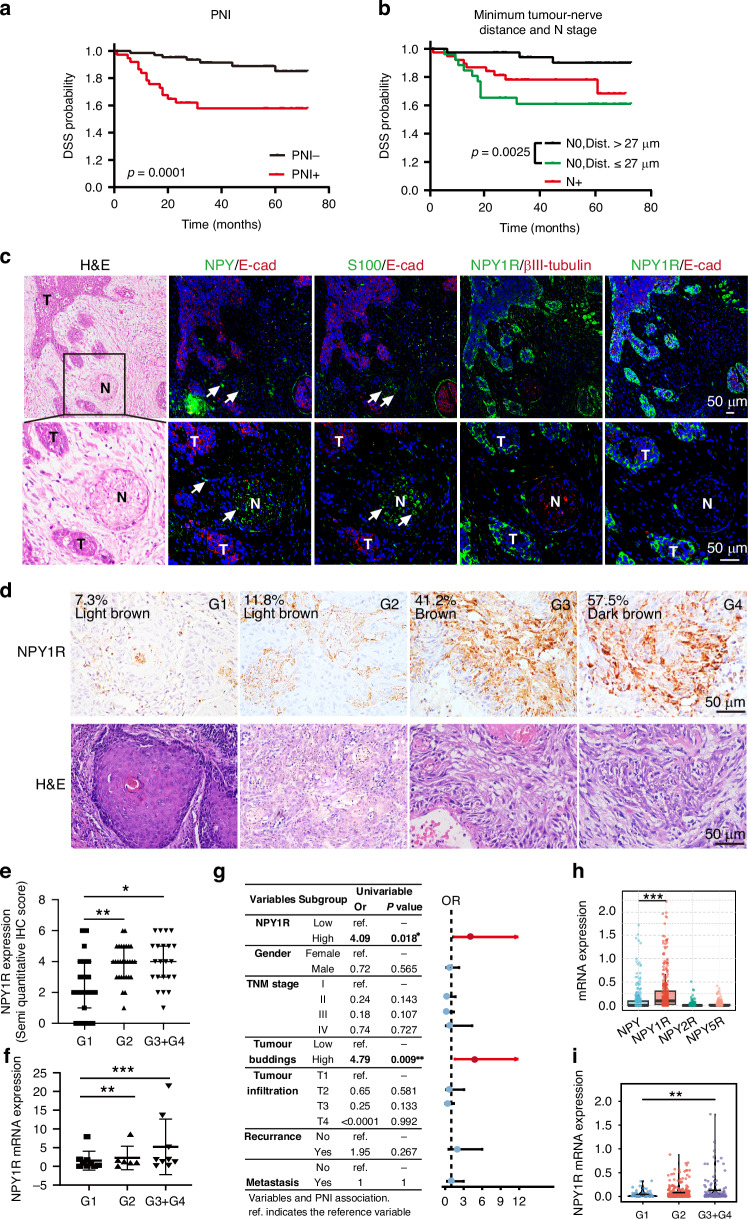
NPY1R is expressed in PNI and correlated with malignancy of OSCC. **a** Kaplan–Meier analysis of overall survival in patients with (PNI + , n = 37) and without (PNI-, n = 66) perineural invasion. **b** Disease-specific survival (DSS) analysis of patient subgroups: N0 (no metastasis) with Dist >27 µm (n = 39), N0 with Dist ≤27 µm (n = 26), and N+ (with metastasis, n = 38). **c** Representative images of H&E and multiplex immunofluorescence (IF) staining for NPY, E-cadherin (E-cad), S-100, βIII-tubulin, and NPY1R in human OSCC tissue (white arrow point to nerve, N, nerve; T, tumour). Scale bar = 50 µm. **d**, **e** Immunohistochemical (IHC) analysis of NPY1R protein expression in OSCC tissues of different pathologic grades: G1 (n = 21), G2 (n = 29), and G3 + G4 (n = 22). Scale bar = 50 µm. **f** Relative mRNA expression levels of NPY1R in OSCC tissues of different grades as determined by qRT-PCR: G1 (n = 9), G2 (n = 6), G3 + G4 (n = 9). **g** Univariate analysis evaluating the relationship between NPY1R expression (by IHC) and clinicopathological parameters of malignant behaviour in OSCC. **h** mRNA expression levels of NPY, NPY1R, NPY2R, and NPY5R in the TCGA-HNSCC cohort (n = 502). **i** Correlation of NPY1R mRNA expression levels with pathologic grade in the TCGA-HNSCC cohort: G1 (n = 62), G2 (n = 301), G3 + G4 (n = 121).

PNI is defined as the tumour invasion close to the nerve or within any of the three layers of the nerve sheath [20, 21]. Consistent with previous reports [14, 22], we found that PNI and the close tumour-nerve distances of less than 27 μm were associated with poor survival in OSCC patients (Fig. 1a, b, Supplementary Fig. 1a, b). To assess whether NPY signalling is involved in PNI, we performed co-IF staining on serial sections of OSCC. Neural markers S100 and βIII tubulin were used to identify nerves and E-cadherin to delineate tumour cells, and the specificity of the antibodies was shown in supplementary Fig. 1c, d. we observed that NPY immunoreactivity was not only confined to nerve fibres but also present within the PNI microenvironment, particularly prominent at the invasive front, where the tumour cells frequently bud from aggressive tumours. Correspondingly, NPY1R was expressed in the plasma membrane of tumour cells, with the highest levels observed at the invasive front or in budding tumour clones close to both the nerve and the NPY-rich areas (Fig. 1c). Notably, tumour cells at the invasive front or within budding clones typically exhibit more malignant features [16, 23]. To investigate whether NPY signalling correlates with the malignancy of OSCC, we evaluated the expression of NPY1R in tumour tissue by IHC staining on tissue microarray (TMA) sections, with a panel of 72 cases of OSCC. NPY1R expression is significantly higher in the high malignant grades (G3 + G4) (Fig. 1d, e). The high expression of NPY1R was significantly correlated with gender, pathological grade, TNM stage, lymphatic metastasis, PNI, and poor survival (Table 1). Detection of mRNA with the fresh clinical biopsies by qRT-PCR also confirmed NPY1R significantly increased in the higher grades of OSCC (Fig. 1f). In addition, the expression of NPY1R were associated with tumour budding that is a histopathological maker of malignant invasion (Fig. 1g). The TCGA database [18] with head and neck squamous cell carcinoma (HNSCC) was also applied to analyze the NPY system. NPY has five receptors in mammals but preferentially binds to Y1, Y2, and Y5 [24]. It showed that NPY and NPY1R are the dominant genes expressed in HNSCC, and NPY1R significantly increases in the high grade of HNSCC (Fig. 1h, i), which is consistent with our data. Taken together, these data indicated that NPY is diffused in the PNI area, and the expression of NPY1R increases in the malignant OSCC.

**Table 1:** Correlation between NPY1R expression and clinicopathological characteristics in patients with OSCC (n = 72).

Clinicopathological	Cases(%)	NPY1R expression		*p*-value
characteristics	n = 72	high	low	
Age(years)				>0.9999
<50	11(15.3%)	6	5	
≥50	61(84.7%)	32	29	
Gender				0.0061**
Female	24(33.3%)	7	17	
Male	48(66.7%)	31	17	
Tumour infiltration				0.2927
T1	9(12.5%)	2	7	
T2	32(44.4%)	18	14	
T3	18(25%)	10	8	
T4	13(18.1%)	8	5	
Pathologic grade				0.0303*
G1	21(29.2%)	6	15	
G2	29(40.3%)	19	10	
G3 + G4	22(30.5%)	13	9	
TNM stage				0.0058**
Ⅰ	7(9.7%)	1	6	
Ⅱ	20(27.8%)	7	13	
Ⅲ	17(23.6%)	9	8	
Ⅳ	28(38.9%)	21	7	
Lymphatic metastasis				0.0010**
N0	38(52.8%)	13	25	
N1-N3	34(47.2%)	25	9	
PNI				0.0275*
No	54(75%)	21	33	
Yes	18(25%)	13	5	
Tumour recurrence				0.592
Positive(+)	17(23.6%)	10	7	
Negative(−)	55(76.4%)	28	27	
Survival				0.0071**
Dead	14(19.4%)	12	2	
Survived	58(80.6%)	26	32	

*p < 0.05, **p < 0.01. Low expression refers to IHC score <4, high expression refers to IHC score ≥4.

### NPY signalling cross-talks with TGFβ signalling in PNI circumstances

**Fig. 2:**
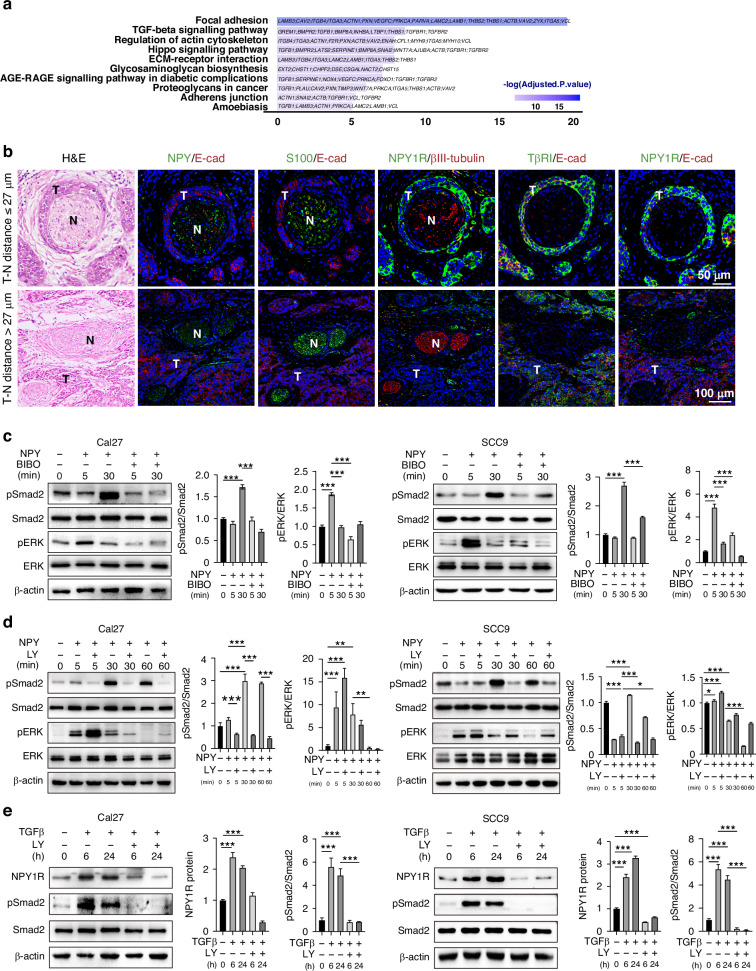
NPY signalling cross-talks with TGFβ signalling in PNI circumstances. **a** Enrichment analysis of KEGG pathways for PNI-related genes using the TCGA-HNSCC database. **b** Representative images of H&E and multiplex immunofluorescence (IF) staining for NPY, E-cadherin (E-cad), S-100, βIII-tubulin, TβRI, and NPY1R in human OSCC tissue (N, nerve; T, tumour) to show tumour-nerve(T-N) interaction. T-N distance ≤ 27 µm (upper panel) and T-N distance > 27 µm (lower panel). Scale bar = 50 μm. **c**–**e** Western blot analysis of pSmad2, total Smad2, pERK, total ERK, and NPY1R in Cal27 and SCC9 cells treated with NPY, the NPY1R antagonist BIBO3304 (BIBO), TGFβ, or the TβRI inhibitor LY2157299 (LY) for the indicated durations. Relative protein levels, quantified using ImageJ software, are presented in the graphs.

To understand the complexity of the PNI microenvironment, we further used the TCGA database to analyze the differentially expressed genes (DEGs) in the cases with or without nerve invasion [18]. The upregulated genes in the samples with nerve invasion were applied for gene enrichment analysis, showing involvement in the function of focal adhesion, TGFβ signalling, and regulation of the actin cytoskeleton (Fig. 2a). TGFβ signalling is one of the main regulators in cell adhesion and cytoskeleton, which suggests that TGFβ might be implicated in PNI circumstances. To investigate this, co-IF staining was applied to detect TβRI and NPY system in OSCC tumour tissues, since TβRI plays a key role in activating the downstream Smad and non-Smad signalling pathways [25]. Interestingly, the tumour cells invading the nerves with the minimum tumour-nerve distance (≤27 μm) expressed a higher level of NPY1R (Fig. 2b upper panel). In contrast, the tumour cells with an increased tumour-nerve distance (>27 μm) expressed a very low level of NPY1R (Fig. 2b lower panel). Notably, TβRI was highly expressed in the cancer cells that expressed a high level of NPY1R in the PNI (Fig. 2b). OSCC cell lines Cal 27 and SCC9 were applied to investigate the downstream signalling. As expected, NPY activated its classical downstream ERK 1/2, and it was blocked by NPY1R inhibitor BIBO3304. Interestingly, NPY also induced the phosphorylation of Smad2 (Fig. 2c), indicating NPY activated TGFβ canonic signalling pathway. This activation is NPY1R-dependent since the NPY1R inhibitor blocked it. In the canonical TGFβ signalling pathway, TβRI phosphorylates Smad2 in C-termini at a conserved motif (Ser-Ser-X-Ser) [26]. Therefore, TβRI kinase inhibitor Galunisertib LY 2157299 was applied and found to completely block NPY-induced activation of Smad2, suggesting this activation is TβRI-dependent (Fig. 2d). A long-term observation in OSCC cells showed this activation could maintain for 24 h, but the peak occurred after stimulation for 30 to 60 min (Supplementary Fig. 2a). In addition to this cross-activating signalling, we also found TGFβ1 treatment induced the expression of NPY1R (Fig. 2e, Supplementary Fig. 2b), which function as feed-forward regulation for this reciprocal regulation. These data suggest that NPY cross talked with TGFβ signalling in the PNI microenvironment.

### NPY promotes the proliferation and invasion of OSCC cells

**Fig. 3:**
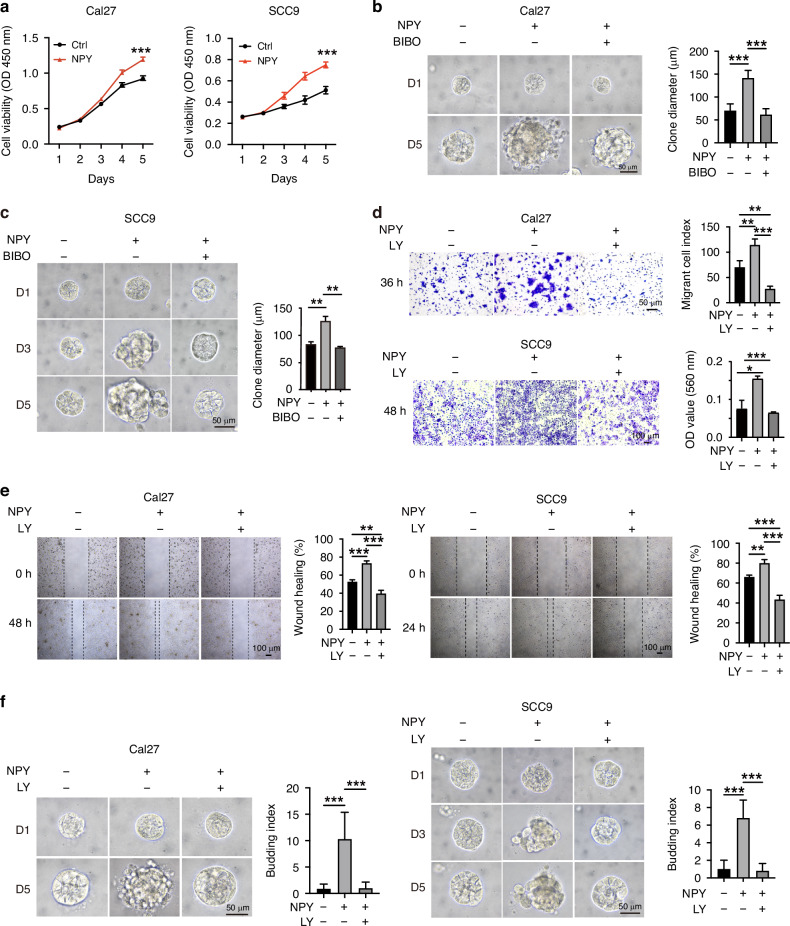
NPY promotes the proliferation and invasion of OSCC cells. **a** Cell proliferation of Cal27 and SCC9 cells treated with NPY, as measured by CCK-8 assay. **b**, **c** 3D sphere formation capacity of Cal27 and SCC9 cells following stimulation with NPY and/or the NPY1R antagonist BIBO for 5 days. Scale bar = 50 μm. The quantification of sphere number or size is shown. **d** Cell invasion of Cal27 and SCC9 cells treated with NPY and/or the TβRI inhibitor LY2157299 (LY), as assessed by Transwell invasion assay. **e** Cell migration of Cal27 and SCC9 cells treated with NPY and/or LY2157299 (LY), evaluated by wound healing assay. Scale bar = 100 μm. **f** Tumour budding in the 3D sphere model of Cal27 and SCC9 cells treated with NPY and/or LY2157299 (LY). Scale bar = 50 μm.

NPY was reported to be involved in the regulation of cell proliferation both in physical and tumour conditions [12, 27]. CCK8 assay showed that NPY significantly induced the proliferation of OSCC cells (Fig. 3a). A 3D sphere culture showed that NPY promoted the formation of larger spheres, while this effect was blocked by using NPY1R kinase inhibitor BIBO3304 (Fig. 3b, c). The proliferation marker Ki67 was stained in these spheres, confirming that NPY promotes cell proliferation while NPY1R inhibitor prevented this effect (Supplementary Fig. 3a). These data suggest that NPY regulates the proliferation of OSCC cells, which is NPY1R-dependent.

Given that NPY cross-activates Smad2 signalling, a key regulator of epithelial-mesenchymal transition (EMT) in cancer invasion, we next evaluated the impact of NPY on cell migration and invasion. We found NPY did promote migration and invasion of OSCC cells, but it was prevented by TβRI kinase inhibitor LY 2157299 (Fig. 3d, e). Interestingly, in a 3D sphere assay, bigger spheres with more budding colonies were observed in the NPY treatment group, while the spheres with fewer buds formed in the LY 2157299 treatment or control groups (Fig. 3f). These data indicate that the crosstalk of NPY with TGFβ signalling promotes the migration and invasion of OSCC cells.

### NPY1R mediates the proliferation and invasion of OSCC cells

**Fig. 4:**
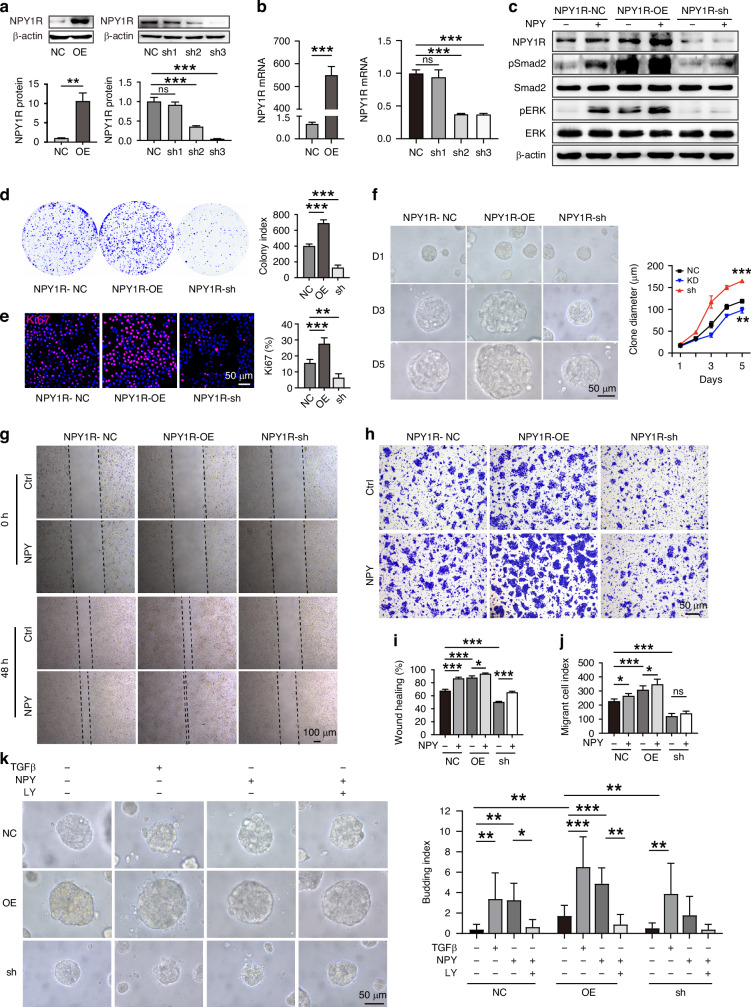
NPY1R mediates the proliferation and invasion of OSCC cells. **a** Lentivirus-based constructs for NPY1R overexpression (NPY1R-OE), knockdown with three distinct shRNAs (NPY1R-sh1, sh2, sh3), and a negative control (NPY1R-NC) in Cal27 cells. **b** Relative mRNA expression of *NPY1R* in the generated stable cell lines, confirming overexpression and knockdown efficiency. **c** Western blot analysis of NPY1R, pSmad2, total Smad2, pERK, and total ERK in NPY1R-OE, NPY1R-sh, and NPY1R-NC cell lines with or without NPY stimulation for 24 h. **d** Colony formation assay of the stable cell lines. **e** Representative immunofluorescence images of Ki67 (proliferation marker, red) and Hoechst (nuclei, blue) in the stable cell lines. Scale bar = 50 μm. **f** 3D sphere formation capacity of the stable cell lines. Scale bar = 50 μm. Wound healing migration assay (**g**) and its quantification (**i**) for the stable cell lines treated with NPY. Transwell migration assay (**h**) and its quantification (**j**) for the stable cell lines treated with NPY. **k** Sphere budding assay of the stable cell lines evaluated upon TGFβ1 or NPY stimulation with or without the TβRI inhibitor LY2157299. Scale bar = 50 μm.

To further elucidate if NPY crosstalk signalling is involved in OSCC progression, we manipulated NPY1R expression using a lentivirus-based vector system to generate knockdown (NPY1R-sh) or overexpressed (NPY1R-OE) OSCC cell lines (Fig. 4a, b, Supplementary Fig. 4a). In NPY1R- OE cells, pSmad2 and pERK were markedly increased upon NPY stimulation, whereas their activation was diminished in NPY1R-sh cells (Fig. 4c). This indicated the cross-activation of Smad2 was NPY1R-dependant. Interestingly, even in the absence of exogenous NPY, NPY1R overexpression alone led to elevated basal levels of pSmad2 and pERK, suggesting that NPY1R may exhibit constitutive activity when highly expressed (Fig. 4c). Ligand-independent constitutive activity is often reported in GPCRs, particularly when receptors are overexpressed, e.g., β2-adrenergic, 5-HT2C receptors [28–30]. We further elucidated the role of NPY1R in this crosstalk signalling. In the CCK8 assay, overexpression of NPY1R promoted OSCC cell proliferation, while knocked down of NPY1R inhibited cell proliferation (Supplementary Fig. 4b, c). Compared with NPY1R-sh cells, the NPY1R-OE cells formed more colonies in the colony assay (Fig. 4d, Supplementary Fig. 4d). More Ki67^+^ cells were detected in NPY1R-OE cells than in NPY1R-sh cells (Fig. 4e). In the 3D sphere formation assay, NPY1R-OE cells generated larger spheres compared with NPY1R-sh cells (Fig. 4f), supporting the role of NPY signalling in promoting OSCC cell proliferation.

These cells were also used to evaluate the invasive capacity. The scratching assay showed that the NPY1R-OE cells healed the wound earlier than the NPY1R-sh cells (Fig. 4g), and the transwell assay showed the migration capacity increased in NPY1R-OE cells but decreased in NPY1R-sh cells upon NPY treatment (Fig. 4h, Supplementary Fig. 4e). The quantification is shown in Fig. 4i, j. Tumour budding or sprouting represents a phenotype of invasiveness. It has recently been recognised as a predictive diagnostic marker [23, 31]. Using a sphere budding model [16], we observed TGFβ1 or NPY stimulation promoted small colonies budding from the primary OSCC spheres. This was more obvious in NPY1R-OE cells since they formed bigger spheres than the control NPY1R-NC and NPY1R-sh cells. Interestingly, NPY-induced tumour budding was inhibited with TβRI kinase inhibitor LY 2157299 treatment (Fig. 4k). This indicated NPY promotes invasive tumour budding by cross-talking with TGFβ signalling. NPY1R might facilitate TβRI to activate Smad2, and we observed that the overexpression of NPY1R enhanced TGFβ-induced pSmad2, while knockdown of NPY1R reduced the level of pSmad2, which suggests NPY1R mediates the activation of Smad2 (Supplementary Fig. 4f). Taken together, we conclude that NPY-induced cell proliferation is NPY1R-dependant, and the crosstalk of NPY and TGFβ signalling mediated cell invasion.

### NPY1R promotes tumour progression in an orthotopic tongue xenograft model

**Fig. 5:**
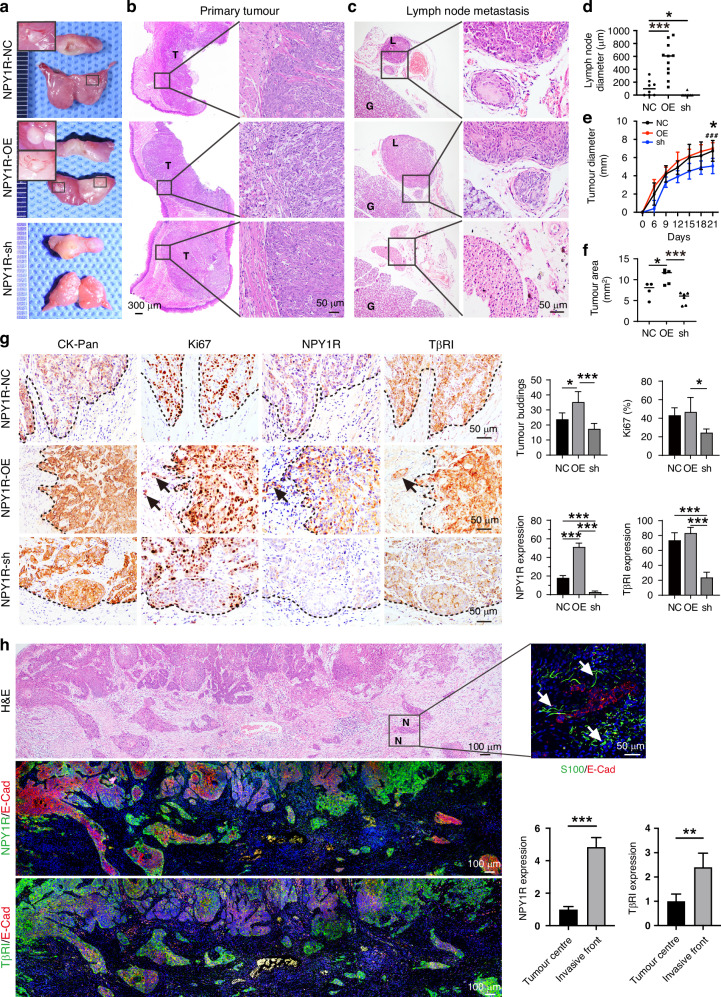
NPY1R promotes tumour progression in an orthotopic tongue xenograft model. **a** Pictures of the orthotopic tongue xenograft mouse model to show primary tumours and sentinel lymph nodes collected from three stable Cal27 strains groups (NPY1R-NC, NPY1R-OE, and NPY1R-sh;n = 5 mice per group). H&E staining of primary tongue tumours (**b**) and lymph nodes (**c**) from the xenograft model (T, tumour; G, gland; L, lymph node metastasis). Quantification of lymph node sizes (**d**), primary tumour sizes (**e**), and tumour area (**f**). **g** Representative IHC staining and quantification of CK-pan (tumour cells), Ki67 (proliferation), NPY1R, and TβRI at the tumour invasion front. Tumour buddings (<5 cells). Scale bar = 50 μm. **h** Panoramic views of NPY1R and TβRI IHC staining in human OSCC tissue sections. Protein expression levels at the tumour invasive front versus the tumour centre were quantified using ImageJ software.

To further investigate the role of NPY1R in OSCC progression in vivo, we applied an orthotopic xenograft model. The NPY1R-NC, NPY1R-OE, and NPY1R-sh were implanted into the tongue of the nude mouse respectively. NPY1R-OE developed into large tumours, while NPY1R-sh cells formed smaller tumours, comparing the control groups (Fig. 5a). Notably, NPY1R OE and the control NC cells metastasised into adjacent regional lymph nodes (Fig. 5a–c). Comparing the control groups, NPY1R OE cells even invade into the neighbouring muscles, blood vessels, and bone (Supplementary Fig. 5a–c). More metastatic lymph nodes were detected in the NPY1R-OE group, compared with NPY1R-sh group (Fig. 5c, d). Consistent with those findings in vitro, overexpression of NPY1R promoted tumour growth in the orthotopic model (Fig. 5e, f). Tumour budding predicts invasiveness and unfavourable survival, associated with lymph node metastasis and PNI [23]. We next evaluated the tumour budding in the invasive front and detected more buds in the NPY1R-OE tumours, where more Ki67-positive cells were detected (Fig. 5g). Interestingly, NPY1R was expressed in the tip of the invasive front where TβRI was also highly expressed (Fig. 5g). To confirm this finding, we examined the OSCC patients’ samples by co-IF staining. With a panoramic picture, the tumour buddings were found apparently in the invasive front, and NPY1R and TβRI were highly expressed in the tumour buds (Fig. 5h). These data suggest NPY1R promotes the invasiveness of OSCC, and TGFβ signalling is involved in this event.

### NPY1R facilitates PNI occurrence

**Fig. 6:**
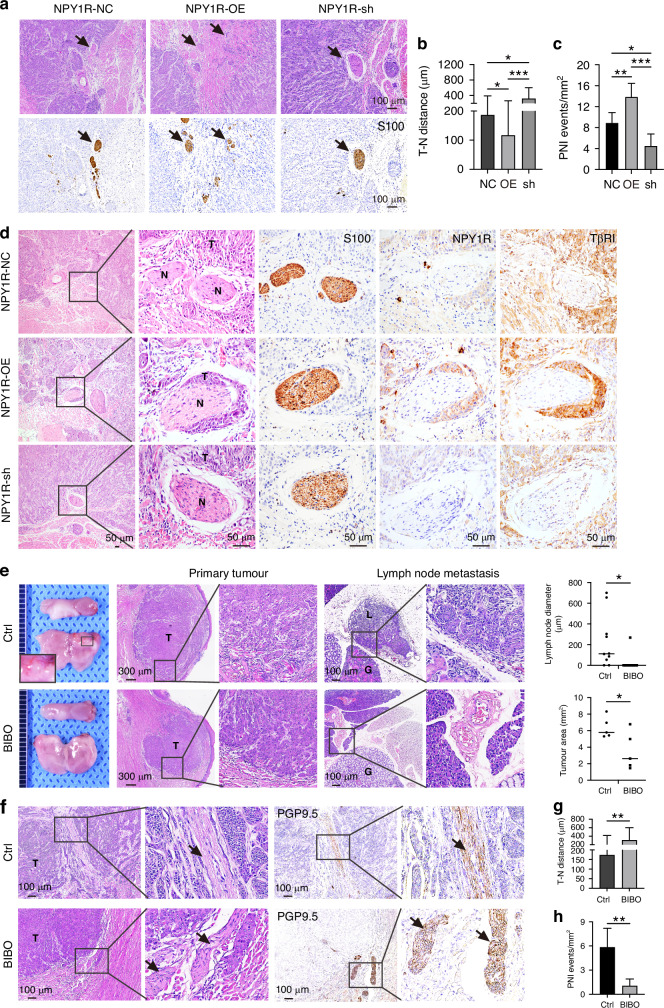
NPY1R facilitates PNI occurrence. **a**–**c** Serial sections of tumours from three groups of orthotopic xenograft model(NPY1R-NC, NPY1R-OE, and NPY1R-sh) stained with H&E and for S100 (a nerve marker) to evaluate tumour-nerve distance and perineural invasion (PNI) events count. Black arrows indicate nerves. Scale bar = 100 μm. **d** Representative images of H&E and IHC staining for S100, NPY1R, and TβRI in tumours from the xenograft model. Scale bar = 50 μm. **e** NTG mice of orthotopic xenograft model(Cal27 cells) treated with the NPY1R inhibitor BIBO3304 (BIBO) or control (n = 5 mice per group).H&E staining of primary tumours and lymph nodes. Quantification of lymph node sizes and tumour area for the control and BIBO3304-treated groups is shown. **f**–**h** Representative serial sections of tumours stained with H&E and for the pan-neuronal marker PGP9.5 to evaluate tumour-nerve(T-N) distance and PNI event counts. Black arrows indicate nerves. (N nerve, T tumour, G gland, L lymph node metastasis).

To elucidate the involvement of NPY1R in PNI progression, we examined the PNI in the same anatomic sites in the tumours developed in the orthotopic tongue xenograft model. PNI is an independent prognostic marker in OSCC patients, and the nerve-tumour distance and nerve density are important parameters for prognosis (Fig. 1a, b) [14, 22]. Following the criteria applied in patient samples [32], we found NPY1R-OE tumour cells invaded nerves and showed the minimum tumour-nerve distance, and higher nerve density, significantly different from the control and NPY1R-sh tumours (Fig. 6a–c). Interestingly, those NPY1R OE tumour cells invading nerves also highly expressed TβRI (Fig. 6d), which indicates the connection of NPY signalling with TGFβ signalling in PNI. To further evaluate the translational potential of targeting the NPY1R- TβRI axis, we performed additional in vivo experiments using an NPY1R inhibitor BIBO. NPY1R blockade markedly reduced tumour growth, metastasis, and perineural invasion (Fig. 6e–h). These results highlight the functional relevance of both NPY1R and TβRI in OSCC progression and provide preclinical evidence that their inhibition can attenuate tumour aggressiveness.

**Fig. 7:**
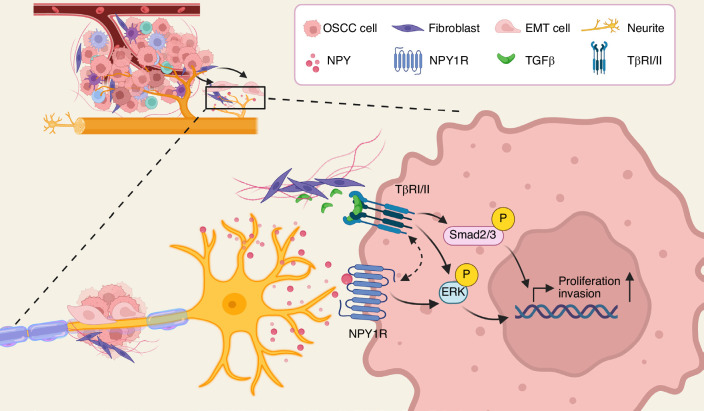
Crosstalk of NPY and TGFβ signalling in PNI of OSCC. This model illustrates the proposed molecular mechanism by which NPY/NPY1R signalling interacts with the TGFβ/Smad & ERK pathway to promote tumour cell migration, invasion, and neural infiltration in OSCC. The schematic was created with BioRender.com.

In summary, we observed enriched NPY localisation surrounding peripheral nerves within PNI regions. Tumour cells appear to exploit this neural signal through high expression of NPY1R, thereby activating downstream ERK1/2 signalling to promote cell proliferation, and cross-activate Smad2 signalling via TβRI to enhance invasion, ultimately driving PNI progression (Fig. 7). These findings identify a previously unrecognised neural-tumour signalling axis and underscore the NPY/NPY1R-TGFβ signalling pathway as a potential therapeutic target for OSCC patients with PNI.

## Discussion

PNI frequently occurs in OSCC progression. In the PNI microenvironment, tumour cells invade the peripheral nerves, or the nerves innervate into the tumour tissue, building up a bidirectional tumour-nerve interaction. Peripheral nerves can sense microenvironmental changes and release neuroactive molecules including neuropeptides, neurotransmitters, and nerve growth factors, maintaining tissue homoeostasis under physiological conditions [33, 34]. In tumours, the nerves are exposed to stress signals and altered nutrient conditions, but the mechanisms by which cancer cells interpret and exploit these neural cues remain incompletely understood. In this context, our study identifies NPY signalling as a critical mediator by which OSCC cells enhance proliferation, invasion, and PNI progression.

NPY belongs to the neuroendocrine peptide family and is widely distributed in the central and peripheral nervous system. In the mammalian brain, NPY is abundantly expressed in the hypothalamic arcuate nucleus, where it integrates metabolic signals via insulin and leptin receptors to regulate energy homeostasis [35–37]. In the peripheral sympathetic nervous system, NPY is co-released with norepinephrine from sympathetic nerve terminals in respond to energy deficiency, fasting, and stress [11, 38]. Beyond the nerve system, NPY is also present in peripheral tissues and expressed by immune cells, including monocyte, lymphocyte and granulocyte, where it modulates immune responses and contributes to shaping the tumour microenvironment. Moreover, certain cancer cells can produce and secrete NPY, and circulating NPY from bloodstream may also reach the tumour site [39, 40]. In OSCC tissues, we observed enriched NPY immunoreactivity within PNI region, particularly at the invasive front where tumour cells adjacent to nerves. These tumour cells expressed high levels of NPY1R, and displayed morphological features associated with greater invasiveness. Previous work by Nisha et al. demonstrated that reduced tumour-nerve distance correlates with poor survival, and that nerves near tumours exhibit stress responses and structural damage, potentially triggering tumour-associated neurogenesis [4, 14]. Given that sympathetic nerves rapidly release norepinephrine under stress, and NPY is co-released as a norepinephrine co-transmitter, it is plausible that elevated NPY in the PNI reflects neural release under tumour-induced stress.

While our data supports a neural origin for NPY in the PNI microenvironment, other sources may contribute depending on tumour type, location, and innervation density. Importantly, we found that TGFβ stimulation upregulates NPY1R in OSCC cells. By upregulating NPY1R, TGFβ enhances the capacity of tumour cells to exploit neuronal NPY signals. TGFβ, as a master regulator in the tumour microenvironment, regulates diverse processes including epithelial-mesenchymal transition, immune evasion, and stromal remodelling. Our finding adds a new layer to its pro-tumorigenic functions, suggesting that TGFβ not only drives classical Smad signalling but also primes tumour cell to sense neural cues through NPY1R. NPY-NPY1R signalling amplifies both Smad2 and ERK activation, resulting in a synergistic drive toward tumour proliferation, invasion, and perineural infiltration.

Tumour cells are known to secrete a variety of factors that can stimulate peripheral nerves and facilitate the release of NPY. Inflammatory cytokines such as interleukin-6 (IL-6) and IL-1β has been shown to enhance neuropeptide release [41]. Tumour-derived neurotrophic factors including NGF, BDNF, and GDNF, induce neuronal sprouting and synaptic activity, thereby promoting neuropeptide release. These tumour-nerve mediators may amplify local NPY availability, further strengthening the NPY-NPY1R axis. How tumour cells sense and respond to neuro cues is a central question. In head and neck cancer, Scanlon et al. reported that galanin (GAL) activates its receptor GALR2 to promote invasion, while GAL released from tumour cells induces neurogenesis [4]. Analogously, our study shows that OSCC cells exploit NPY signals, activating ERK1/2 to promote proliferation and Smad2 signalling to enhance migration and invasion.

Mechanistically, our data reveal that NPY signalling crosstalks with TGFβ in the context of PNI. Transcriptomic profiling of OSCC cases with nerve invasion revealed enrichment of TGFβ-related genes, and co-IF staining confirmed TβRI expression in tumour cells invading nerves in NPY-rich PNI regions. NPY stimulation transiently activates Smad2 in a TβRI-and NPY1R-dependent manner. Given that NPY1R is a G protein-coupled receptor (GPCR), and GPCRs have been reported to transactivate TβRI to engage canonical Smad signalling [42], it is plausible that NPY1R functions similarly. Indeed, the orphan GPCR, GPR50 directly binds TβRI to activate its Ser/Thr kinase activity, and mimics TGFβ-induced responses [43]. Consistent with this model, NPY treatment or NPY1R overexpression induced OSCC migration and invasion in a manner comparable to TGFβ stimulation, while NPY1R knockdown or pharmacological inhibition markedly reduced tumour growth and PNI in vivo. However, whether NPY1R physically interacts with TβRI and the precise molecular cascade by which NPY1R transactivates TGFβ signalling remain to be determined.

In addition to its functional role in vitro and in vivo, our clinical data demonstrated that high NPY1R expression was significantly associated with unfavourable clinicopathological parameters in OSCC, including higher pathological grade, advanced TNM stage, lymph node metastasis, and tumour recurrence (Table 1). These correlations further support the notion that NPY1R is not only a mechanistic driver of perineural invasion but also a marker of tumour aggressiveness. Consistent with findings in other malignancies where NPY receptor upregulation correlates with progression and metastatic behaviour, our results suggest that NPY1R overexpression may stratify OSCC patients with higher risk of recurrence and metastasis. Importantly, this clinical association strengthens the translational potential of targeting NPY1R, as it may serve as both a prognostic biomarker and a therapeutic vulnerability in aggressive OSCC.

Dysregulated NPY receptor signalling is not unique to OSCC. In breast cancer, NPY1R is upregulated under hypoxic conditions and promotes proliferation, migration, and angiogenesis, while Y5R supports tumour cell survival and chemoresistance [44]. Interestingly, co-administration of a Y1R ligand with tariquidar has been explored as a strategy to selectively deliver the drug to cancer cells and improve therapeutic efficacy against multidrug-resistant breast cancer. In prostate cancer, both Y1R and Y5R showed high expression in bone metastasis, indicating NPY system correlates with disease progression [12, 45]. In neuroblastoma, NPY via Y2R stimulates angiogenesis and tumor growth, and via Y5R it enhances metastasis, whereas Y2R antagonists reduce cell proliferation and vascularisation [46, 47]. In liver cancer, TGFβ induces NPY release from the peritumoral hepatocytes and the Y5R expression in invasive carcinoma cells [48]. Conversely, in Ewing sarcoma, NPY can inhibit tumor cell survival and induce apoptosis through Y1R and Y5R signalling [49]. These context dependent effects highlight the receptor- and pathway-specific diversity of NPY activation, and underscore the importance of tumor-specific profiling when considering NPY-targeted therapy.

In conclusion, our study identifies a previously unrecognised NPY/NPY1R- TGFβ crosstalk that drives PNI in OSCC. By upregulating NPY1R, TGFβ sensitises tumor cells to neural NPY signals, which activate ERK1/2 and Smad2 pathways to promote proliferation and invasion and neural infiltration. These findings highlight how OSCC cells hijack neuronal signalling to enhance malignant behaviour and position NPY1R as a promising therapeutic target to disrupt tumor-nerve interactions in aggressive disease.

## Supplementary information

Supplementary figure 1

Supplementary figure 2

Supplementary figure 3

Supplementary figure 4

Supplementary figure 5

Supplementary Figures Legendes

## Data Availability

The datasets used or analyzed for the current study are available from the corresponding author upon reasonable request.
