# Supplementary figures and images for: Crosstalk of NPY and TGFβ orchestrates the signaling to facilitate perineural invasion of oral squamous cell carcinoma
Source: Br J Cancer. 2025 Nov 26;134(3):377–90. doi: 10.1038/s41416-025-03261-5 (PMC12852772; doi:10.1038/s41416-025-03261-5)

**a**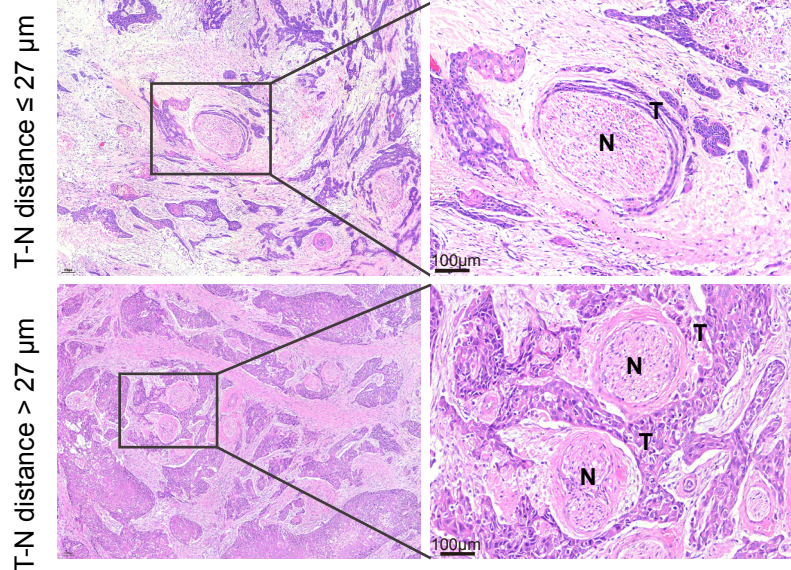**b**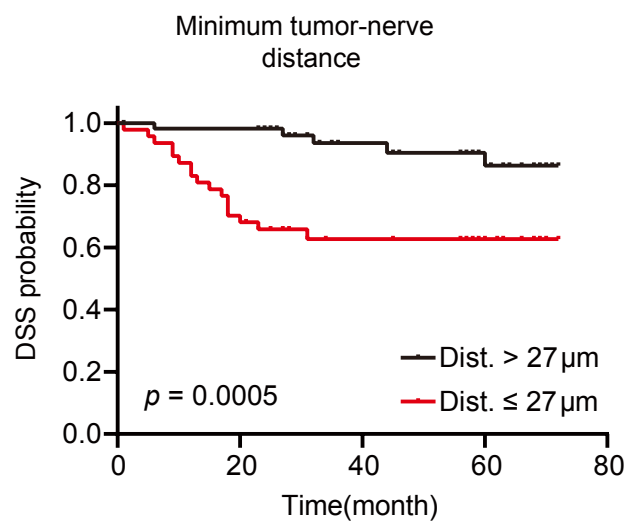**c**

NPY1R Negative Ctrl

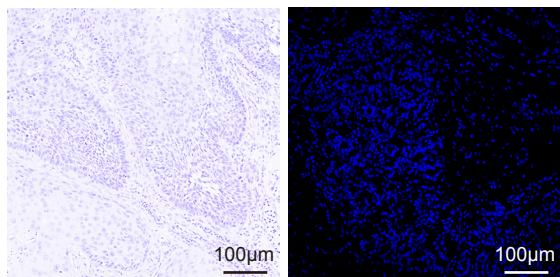**d**NPY  
Negative CtrlNPY Positive Ctrl  
(mouse brain)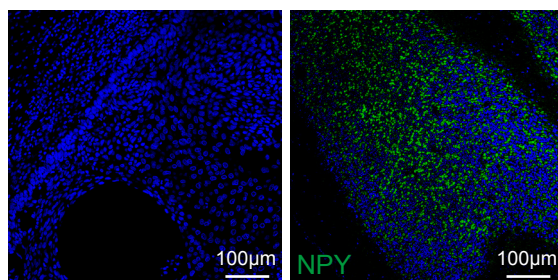

Supplement: Supplementary file 1 — Supplementary figure 1 [file 41416_2025_3261_MOESM1_ESM.pdf]

**a**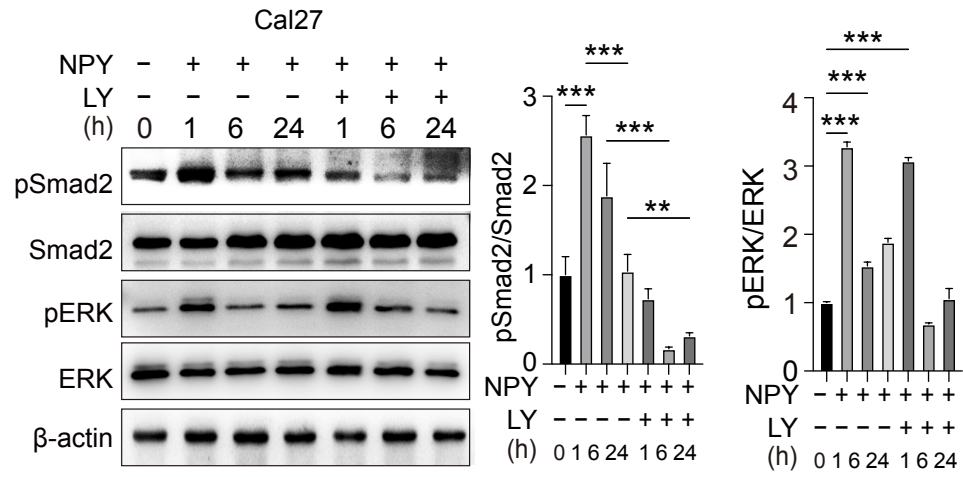**b**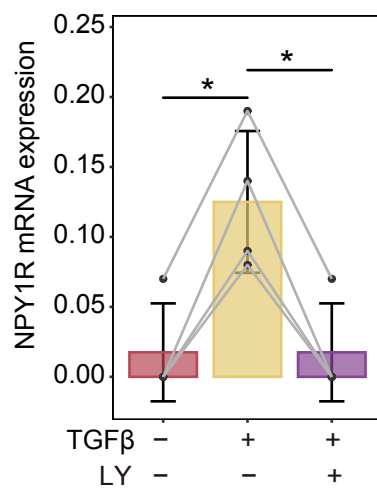

Supplement: Supplementary file 2 — Supplementary figure 2 [file 41416_2025_3261_MOESM2_ESM.pdf]

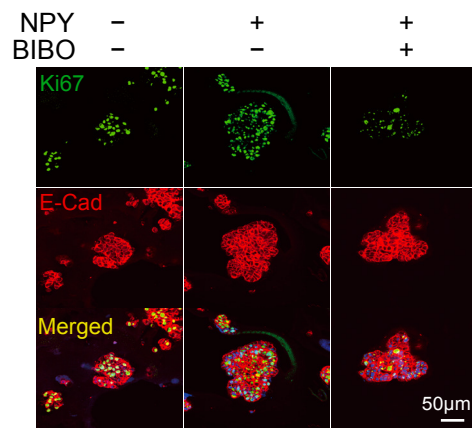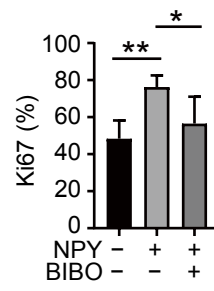

Supplement: Supplementary file 3 — Supplementary figure 3 [file 41416_2025_3261_MOESM3_ESM.pdf]

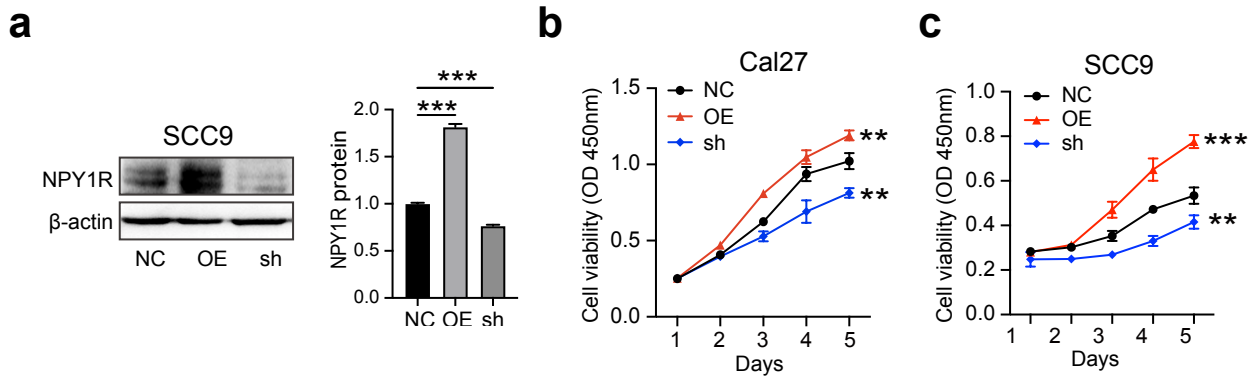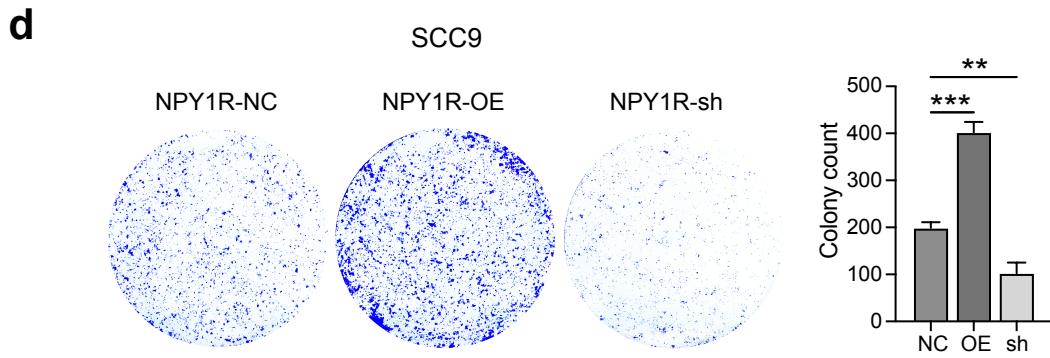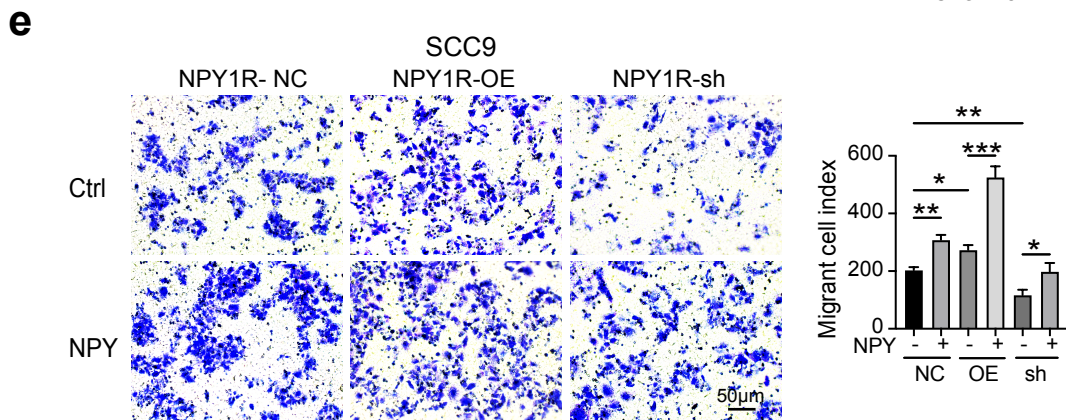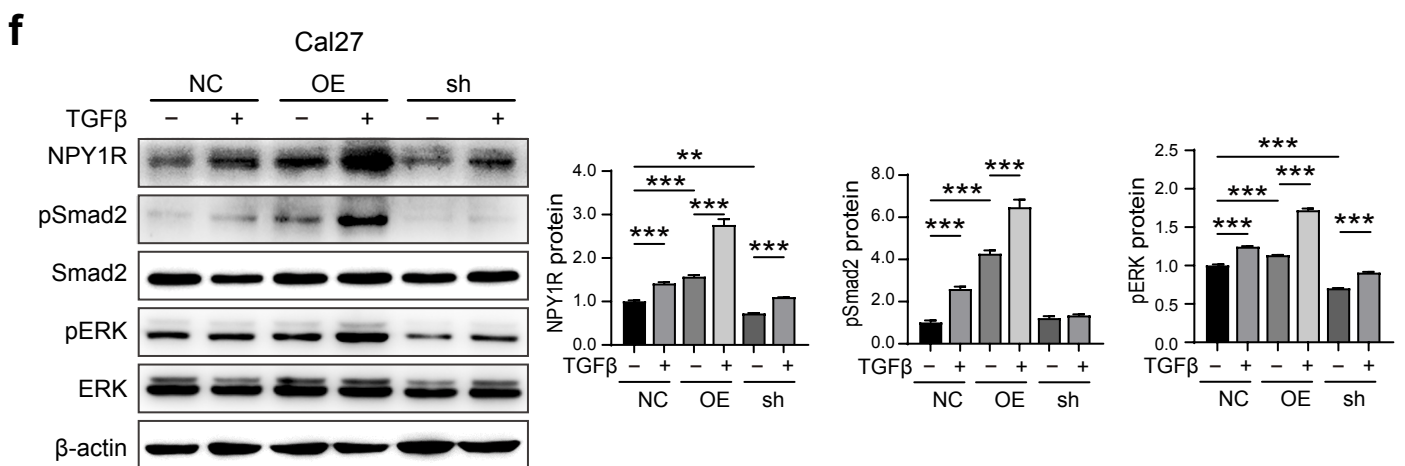

Supplement: Supplementary file 4 — Supplementary figure 4 [file 41416_2025_3261_MOESM4_ESM.pdf]

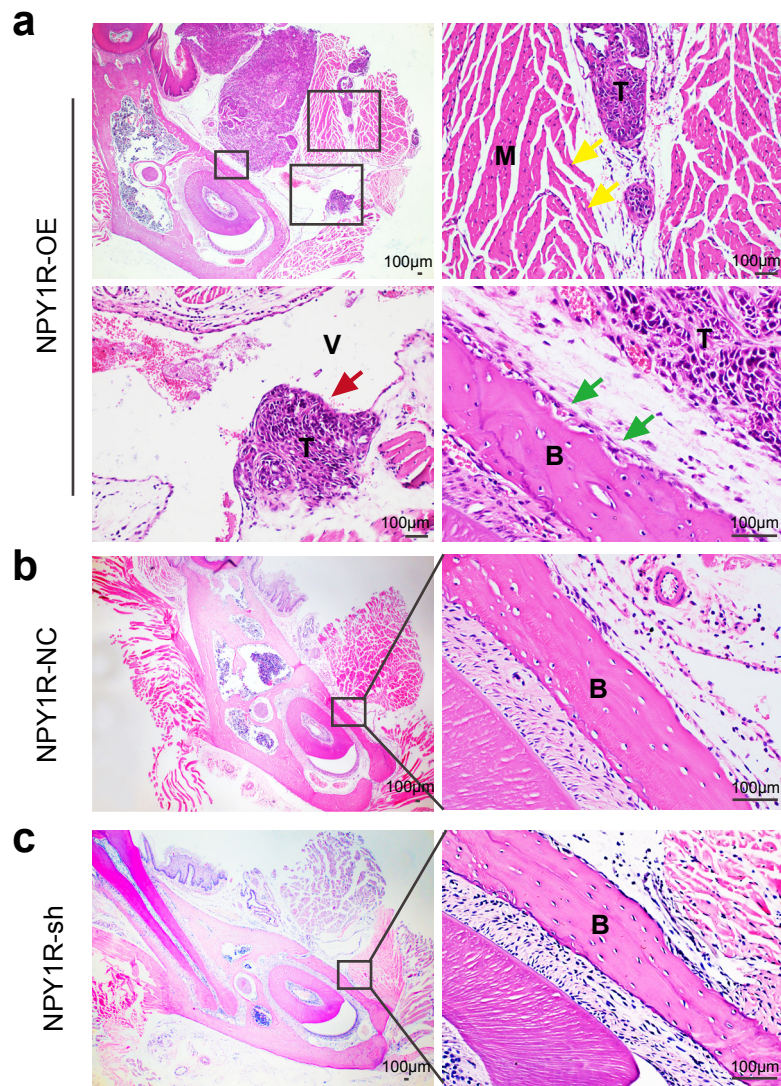

Supplement: Supplementary file 5 — Supplementary figure 5 [file 41416_2025_3261_MOESM5_ESM.pdf]
