# Supplementary material for: Crosstalk of NPY and TGFβ orchestrates the signaling to facilitate perineural invasion of oral squamous cell carcinoma
Source: Br J Cancer. 2025 Nov 26;134(3):377–90. doi: 10.1038/s41416-025-03261-5 (PMC12852772; doi:10.1038/s41416-025-03261-5)
Supplement: Supplementary file 6 — Supplementary Figures Legendes [file 41416_2025_3261_MOESM6_ESM.docx]

**Supplementary Fig. 1**

(a) Representative images illustrating the two modes of PNI: active invasion (upper panel),

defined by a tumor-nerve distance (Dist) ≤ 27 μm, and passive invasion (lower panel),

characterized by a Dist > 27 μm. N, nerve; T, tumor.(b) Kaplan-Meier survival curve comparing

overall survival between patients with Dist ≤ 27 μm (n=47) and those with Dist > 27 μm

(n=56).(c) Negative control for NPY1R immunostaining in an OSCC paraffin section.(d) Assay

specificity controls for NPY immunostaining: negative control in an OSCC section (left panel)

and positive control in mouse brain tissue (right panel). Scale bar = 100 μm (applies to all

panels).

**Supplementary Fig. 2**

(a) Western blot analysis of pSmad2, total Smad2, pERK, and total ERK in Cal27 and SCC9 cells

treated with NPY or the TβRI inhibitor LY2157299 (LY) for the indicated durations. Relative

gray values, quantified using ImageJ software, are shown below the respective bands.

(b) Relative mRNA expression levels of NPY1R in Cal27 cells treated with TGFβ or the TβRI

inhibitor LY2157299 (LY) for 24 hours.

**Supplementary Fig. 3**

(a) Immunofluorescence (IF) staining of Ki67 and E-cadherin in SCC9 tumor spheres treated

with NPY and/or the NPY1R antagonist BIBO3304 (BIBO) for 5 days. Scale bar = 50 μm.

**Supplementary Fig. 4**

(a) Western blot analysis confirming stable NPY1R overexpression (OE), knockdown (sh), or

negative control (NC) in SCC9 cells. (b, c) Proliferation rates of Cal27 (b) and SCC9 (c) cells, as

determined by CCK-8 assay. (d) Colony formation assay of SCC9 cells under the indicated

conditions. (e) Transwell migration assays of SCC9 cells treated with or without

NPY. (f) Western blot analysis of pSmad2, total Smad2, pERK, and total ERK in SCC9 cells with

modulated NPY1R expression following TGFβ stimulation.

**Supplementary Fig. 5**

(a) H&E staining revealed tumor invasion into adjacent muscle (yellow arrow), vasculature(red

arrow), and bone( green arrow) in the NPY1R-OE group (2/5 mice), as indicated by yellow, red, and green arrows, respectively. This invasive phenotype was not observed in the NPY1RNC(

b) or NPY1R-sh control groups(c). Scale bar = 100 μm. T, tumor; B, bone; V, vessel; M,muscle.
